# Bone remodeling in the absence of osteocytes

**DOI:** 10.1016/j.bonr.2026.101959

**Published:** 2026-10-14

**Authors:** Larry J. Suva

**Affiliations:** Department of Veterinary Physiology and Pharmacology, College of Veterinary Medicine and Biomedical Sciences, Texas A&M University, College Station, TX, 77843, United States of America

## Abstract

**Figure:**
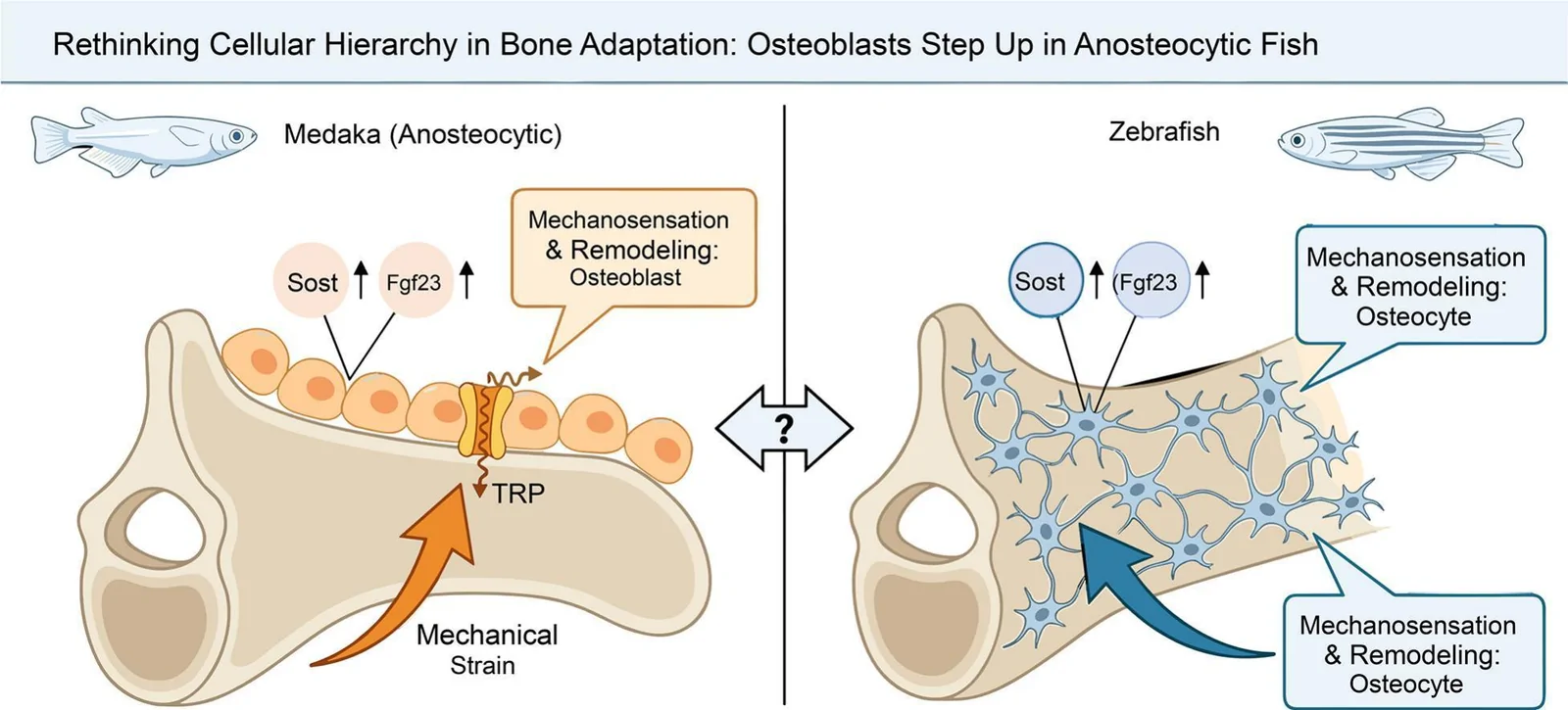

Bone biology has long focused on the osteocyte, once regarded as a cell type with no function but now considered crucial for sensing mechanical strain, orchestrating remodeling, and playing a central role in all aspects of skeletal function (Burger et al., 1995; Lanyon, 1993; Turner and Forwood, 1995; Bonewald and Johnson, 2008). This paradigm, driven by an interest in how bones respond to mechanical loading, particularly how they respond as structures rather than merely accumulations of strain site specific cells stimulated investigation into which cells were responsible (Klein-Nulend et al., 1995; Rawlinson et al., 1996; Skerry et al., 1989). Current dogma holds that osteocytes translate physical forces into biochemical signals, guiding bone structure, adaptation, and maintenance across the lifespan (Robling and Bonewald, 2020; Duncan and Turner, 1995).

However, the existence of anosteocytic fish (Weiss and Watabe, 1979), organisms that lack osteocytes, yet possess normal bone architecture and robust adaptive bone remodeling, poses a direct evolutionary challenge to the view that only osteocytes can serve as mechanical sensors in bone (Atkins et al., 2015). The recent paper, “Maintenance of bone health by osteoblasts in the absence of osteocytes: comparative analysis of gene expression in medaka and zebrafish vertebrae,” (Jayash et al., 2026) investigates this puzzle, shedding light on alternative pathways and refocusing attention on the role of other cells in skeletal maintenance. Indeed, fish lacking osteocytes demonstrate that a response to a strain distribution can be achieved and used to control adaptive bone remodeling by non-osteocytic cells (presumably osteoblasts on the bone surface) without the need for strain information from within the depths of the bone.

The study's significance lies in its fundamental challenge to established assumptions in bone biology. By demonstrating that bones can sense strain and undergo adaptive remodeling in the absence of osteocytes, the authors redefine the cellular repertoire responsible for mechanosensation and bone homeostasis. Rather than follow the canonical mammalian model, they provide evidence that osteoblasts, and not osteocytes, mediate key mechanosensory functions in medaka, an anosteocytic teleost, whereas zebrafish, with osteocytic bone, retain the osteocyte-centric paradigm. This striking divergence underscores the evolutionary flexibility in bone biology and suggests that the mechanisms underpinning bone adaptation may be broader and more plastic than previously appreciated. As a result, researchers are compelled to revisit the cellular hierarchy of bone, to consider the diversity of skeletal strategies across vertebrates, and to re-evaluate some of the foundational assumptions that have governed the field for decades.

There is ample evidence across many years demonstrating that mammalian osteoblast-like cells are mechanosensitive (Damien et al., 1998; Feik et al., 1990; Sen et al., 2009; Owan et al., 1997). More recently, the widespread expression of the mechanosensitive Piezo cation channels suggest that there is a capability for mechanical sensing across many cell types (Wu et al., 2017). Collectively, these findings remind us that mechanosensing in bone can be accomplished by cells other than osteocytes. This current observation has the potential to refocus thinking about how bone evolved to meet the demands of varying mechanical environments, particularly amongst vertebrates inhabiting drastically different niches.

Although this is not the first observation of adaptation in anosteocytic bone, the originality of the research is rooted in the high-resolution molecular techniques used to dissect cell-type-specific gene expression in fish bone. Unlike previous studies that relied on anatomical or broader transcriptomic approaches (Tauer et al., 2024), this work utilized in situ hybridization and single nucleus RNA sequencing (snRNA-seq) to pinpoint the cellular source of critical osteocyte markers, such as sclerostin (SOST) and fibroblast growth factor (FGF23). Interestingly, in medaka, these genes are highly expressed in osteoblasts, not osteocytes, perhaps suggesting an evolutionary transfer of cell function. Additionally, the identification of multiple transcriptional clusters and the enrichment of transient receptor potential (TRP) ion channels highlight alternative pathways that may contribute to bone mechanosensation. Through comparative genomics and functional studies, the authors suggest that osteoblasts in anosteocytic species may have adopted “osteocyte-like” roles, thereby offering a compelling narrative of evolutionary adaptation and cellular versatility. These findings revive the idea (or remind us) that osteoblasts can be mechanosensitive (Duncan and Turner, 1995; Moore et al., 2019), but in the context of anosteocytic fish, this old concept is repositioned as a central principle. The detailed mapping of gene expression, including both established and novel markers of mechanosensitive cells, marks an important milestone for the field.

Clinically, this research opens new avenues for treating bone disorders linked to osteocyte dysfunction or loss, such as osteoporosis, and other metabolic bone diseases (Lanyon and Skerry, 2001; Marino and Bellido, 2024). If mammalian osteoblasts can substitute, at least in part, for absent or dysfunctional osteocytes, then therapies could be designed to enhance osteoblast mechanosensory and homeostatic roles in diseased or aging bone. The elucidation of the TRP channel pathways as possible mechanosensors is particularly significant, as these channels become attractive drug targets for modulating bone adaptation and remodeling. These findings offer hope for regenerating bone, restoring remodeling capacity, or protecting bone mass in situations where osteocyte networks are compromised, such as aging and glucocorticoid-induced osteoporosis. Furthermore, the study may inform innovations in biomaterials and implant design. Mimicking the adaptive mechanisms observed in medaka bone, where mechanosensation and remodeling occur independent of embedded osteocytes, could inspire new strategies for optimizing the integration of orthopedic implants or accelerating bone healing.

Despite the exciting nature of these findings, several important limitations must be acknowledged. First, the snRNA-seq analyses likely included some non-skeletal cell populations, introducing potential confounders and necessitating improved tissue isolation techniques in future studies. Second, the functional roles of many differentially expressed genes, particularly TRP channels and novel osteoblast-expressed markers such as SOST and FGF23, although promising, remain to be validated through targeted gene knockout or knockdown experiments. Without these functional studies, the mechanistic link between gene expression and cell behavior remains speculative. Third, while medaka and zebrafish serve as informative model organisms for studying evolutionary variation, findings may not directly translate to mammals, including humans, without further comparative research. The structural and cellular context of fish bone differs from mammalian bone, and it remains unclear whether similar compensatory mechanisms exist in higher vertebrates. Finally, the study was limited to vertebral bone, leaving open questions about other skeletal compartments and whole-organism physiology. Examination of cranial or fin bones, for example, may yield additional insights into the plasticity and adaptability of bone maintenance mechanisms.

Building on these discoveries, several promising avenues for future research emerge. Functional experiments using gene editing in medaka could directly test the roles of osteoblast-specific expression of osteocyte markers in bone adaptation. Improved single-cell sequencing approaches, minimizing soft tissue contamination, will provide higher resolution maps of skeletal cell populations and their gene expression dynamics. Comparative studies across other teleosts, as well as reptiles, birds, and mammals with variable osteocyte populations, will help trace the evolutionary trajectory of bone mechanosensation. Mechanistic investigations into TRP channel signaling in osteoblasts are warranted. Understanding how these ion channels integrate environmental strain and regulate downstream gene expression may uncover new intervention points. Ultimately, further research should address whether augmenting osteoblast function or mimicking medaka's adaptations can restore bone health or prevent bone pathology in humans.

This article reminds the bone biology field that the maintenance and adaptation of bone is not solely the province of osteocytes. By identifying osteoblasts as key players in mechanosensation and homeostasis in anosteocytic fish, and highlighting alternative mechanosensory pathways, the authors reveal a remarkable evolutionary ingenuity at the core of skeletal tissue. Hopefully, the findings will influence basic science and translational research to consider not just the role of osteocytes but also the osteoblast in mechanotransduction. As the field advances, further explorations inspired by this work will undoubtedly deepen our understanding of bone diversity, adaptation, and therapeutic potential.

## CRediT authorship contribution statement

**Larry J. Suva:** Writing – review & editing.

## Declaration of Generative AI and AI-assisted technologies in the writing process

During the preparation of this work, the author used ChatGPT to suggest an appropriate title for the commentary. The author reviewed and edited the output as needed and takes full responsibility for the content of the published article.

## Declaration of competing interest

The authors declare that they have no known competing financial interests or personal relationships that could have appeared to influence the work reported in this paper.

## Data Availability

No data was used for the research described in the article.

## References

[bb0005] Atkins A., Milgram J., Weiner S., Shahar R. (2015). The response of anosteocytic bone to controlled loading. J. Exp. Biol..

[bb0010] Bonewald L.F., Johnson M.L. (2008). Osteocytes, mechanosensing and Wnt signaling. Bone.

[bb0015] Burger E.H., Klein-Nulend J., Van Der Plas A., Nijweide P.J. (1995). Function of osteocytes in bone--their role in mechanotransduction. J. Nutr..

[bb0020] Damien E., Price J.S., Lanyon L.E. (1998). The estrogen receptor's involvement in osteoblasts' adaptive response to mechanical strain. J. Bone Miner. Res..

[bb0025] Duncan R.L., Turner C.H. (1995). Mechanotransduction and the functional response of bone to mechanical strain. Calcif. Tissue Int..

[bb0030] Feik S.A., Ellender G., Crowe D.M., Ramm-Anderson S.M. (1990). Periosteal response in translation-induced bone remodelling. J. Anat..

[bb3000] Jayash S.N., Dunbar D.R., Guizard S., Furniss J., Ruiz Daniels R., Pitsillides A.A., Staines K.A., Robledo D., Farquharson C., Stephen L.A. (2026). Maintenance of bone health by osteoblasts in the absence of osteocytes: comparative analysis of gene expression in medaka and zebrafish vertebrae. Bone Reports.

[bb0035] Klein-Nulend J., Semeins C.M., Ajubi N.E., Nijweide P.J., Burger E.H. (1995). Pulsating fluid flow increases nitric oxide (NO) synthesis by osteocytes but not periosteal fibroblasts--correlation with prostaglandin upregulation. Biochem. Biophys. Res. Commun..

[bb0040] Lanyon L., Skerry T. (2001). Postmenopausal osteoporosis as a failure of bone's adaptation to functional loading: a hypothesis. J. Bone Miner. Res..

[bb0045] Lanyon L.E. (1993). Osteocytes, strain detection, bone modeling and remodeling. Calcif. Tissue Int..

[bb0050] Marino S., Bellido T. (2024). PTH receptor signalling, osteocytes and bone disease induced by diabetes mellitus. Nat. Rev. Endocrinol..

[bb0055] Moore E.R., Chen J.C., Jacobs C.R. (2019). Prx1-Expressing Progenitor Primary Cilia Mediate Bone Formation in response to Mechanical Loading in Mice. Stem Cells Int..

[bb0060] Owan I., Burr D.B., Turner C.H., Qiu J., Tu Y., Onyia J.E., Duncan R.L., New Collective A. (1997). Mechanotransduction in bone: osteoblasts are more responsive to fluid forces than mechanical strain. Am. J. Phys..

[bb0065] Rawlinson S.C., Pitsillides A.A., Lanyon L.E. (1996). Involvement of different ion channels in osteoblasts' and osteocytes' early responses to mechanical strain. Bone.

[bb0070] Robling A.G., Bonewald L.F. (2020). The Osteocyte: New Insights. Annu. Rev. Physiol..

[bb0075] Sen B., Styner M., Xie Z., Case N., Rubin C.T., Rubin J. (2009). Mechanical loading regulates NFATc1 and beta-catenin signaling through a GSK3beta control node. J. Biol. Chem..

[bb0080] Skerry T.M., Bitensky L., Chayen J., Lanyon L.E. (1989). Early strain-related changes in enzyme activity in osteocytes following bone loading in vivo. J. Bone Miner. Res..

[bb0085] Tauer J.T., Thiele T., Julien C., Ofer L., Zaslansky P., Shahar R., Willie B.M. (2024). Swim training induces distinct osseous gene expression patterns in anosteocytic and osteocytic teleost fish. Bone.

[bb0090] Turner C.H., Forwood M.R. (1995). What role does the osteocyte network play in bone adaptation?. Bone.

[bb0095] Weiss R.E., Watabe N. (1979). Studies on the biology of fish bone. III. Ultrastructure of osteogenesis and resorption in osteocytic (cellular) and anosteocytic (acellular) bones. Calcif. Tissue Int..

[bb0100] Wu J., Lewis A.H., Grandl J. (2017). Touch, Tension, and Transduction - The Function and Regulation of Piezo Ion Channels. Trends Biochem. Sci..

